# The Role of Physical Resilience in Patient Satisfaction Following Total Knee Replacement

**DOI:** 10.1093/geroni/igaf122.1058

**Published:** 2025-12-31

**Authors:** Qian-Li Xue, Thomas Laskow, Mallak Alzahrani, Karen Bandeen-Roche, Ravi Varadhan, Jeremy Walston, Frederick Sieber

**Affiliations:** Johns Hopkins University, Baltimore, Maryland, United States; Johns Hopkins University, Baltimore, Maryland, United States; Johns Hopkins University, Baltimore, Maryland, United States; Johns Hopkins Center on Aging and Health, Baltimore, Maryland, United States; Johns Hopkins University, Baltimore, Maryland, United States; Johns Hopkins School of Medicine, Baltimore, Maryland, United States; Johns Hopkins University, Baltimore, Maryland, United States

## Abstract

Predicting patient satisfaction after total knee replacement (TKR) remains a challenge in clinical care. While preoperative predictors are ideal, early post-surgical measures are critical for identifying intervention targets to improve long-term satisfaction. This study examined six-month trajectories of whole-body physical function and their relationships with satisfaction at 12 months in 101 patients aged 60+. Physical function was assessed at baseline, 1 month, and 6 months using the Short Physical Performance Battery and the SF-36 Physical Component Score; fatigue was self-reported using the Pittsburgh Fatigability Scale-Physical Subscale. Latent profile analysis identified distinct trajectory patterns, and logistic regression assessed their associations with high vs. lower satisfaction after adjusting for age and body mass index. Decision curve analysis (DCA) evaluated whether physical function trajectories vs. baseline measures can better guide post-TKR rehabilitation decisions compared to treating everyone or treating no one. At 12 months, 79% (n = 80) reported high satisfaction. Distinct trajectory patterns over six months were identified across all three measures, with the most favorable trajectory for SPPB, PFS, and PCS was associated with 12.6, 8.4-, and 9.8-times greater odds of high satisfaction, respectively, compared to the least favorable trajectory (P < 0.0.3 for all). DCA showed that trajectory-based decision-making provided greater net benefit than baseline measures when the risk threshold for intervention was within the range of 20–60% for SPPB, 14–35% for PFS, and 13–43% for PCS. These findings highlight the value of monitoring early post-surgical physical function trajectories to optimize patient-centered rehabilitation and improve long-term TKR satisfaction.

